# Case report: A frameshift mutation in CLCN2-related leukoencephalopathy and retinopathy

**DOI:** 10.3389/fgene.2023.1278961

**Published:** 2023-11-09

**Authors:** Yizhe Cheng, Xinyu Liu, Limei Sun, Xiaoyan Ding

**Affiliations:** State Key Laboratory of Ophthalmology, Zhongshan Ophthalmic Center, Sun Yat-sen University, Guangzhou, China

**Keywords:** CLCN2 gene, leukoencephalopathies, retinal degeneration, magnetic resonance imaging, optical coherence tomography

## Abstract

**Background:** Leukoencephalopathy and visual impairment have been linked to loss-of-function mutations in the *CLCN2* gene (MIM #600570). However, the ocular features caused by the *CLCN2* mutations remain poorly understood and seldom reported. This study aims to present a novel mutation and characterize the ocular phenotype in a Chinese female diagnosed with *CLCN2*-related leukoencephalopathy (CC2L), also known as leukoencephalopathy with ataxia (LKPAT; MIM #615651).

**Case presentation:** A 20-year-old Chinese female presented with bilateral blurred vision persisting for 2 years, which had worsened over the past 6 months. Ophthalmologic examination revealed bilateral post-capsular cataracts, macular retinal atrophy, and peripheral retinal pigmentation. Swept-source optical coherence tomography (SS-OCT) showed bilateral choroidal capillary atrophy, loss of the outer retinal layer, and a novel noteworthy sign of vacuole-like vitreoretinopathy. Cranial magnetic resonance imaging confirmed leukoencephalopathy. Genetic testing identified a novel homozygous pathogenic c.1382_1386del (p.P461Lfs*13) mutation in exon 13 of the CLCN2 gene.

**Conclusion:** This case report expands the knowledge of CLCN2 mutations and their associated ocular manifestations in patients with CC2L. The identified ophthalmic features may serve as crucial indicators for early diagnosis in individuals with CC2L, especially in the absence of evident neurological symptoms.

## Introduction

*CLCN2*-related leukoencephalopathy (CC2L), also known as leukoencephalopathy with ataxia (LKPAT; MIM #615651), is a rare autosomal recessively inherited disease caused by *CLCN2* mutations. First reported by Depienne in 2013, CC2L is characterized by cerebellar ataxia, recurrent headache, intention tremor, speech impediments and memory decline (Depienne et al., 2013). The *CLCN2* gene (MIM #600570) encodes for the chloride channel 2 (ClC-2) protein, a two-pore homodimeric, voltage-gated Cl^−^ channel found in various organs and tissues, including the brain, testis, colon, and retina (Zúñiga et al., 2004).

Disruption of ClC-2 could lead to fluid accumulation, resulting in intramyelinic edema (Jeworutzki et al., 2012). Of note, patients with CC2L not only exhibit neurologic symptoms but also manifest visual impairment, including retinal degeneration, optic neuropathy, retinoschisis and other abnormal changes. However, reports of CC2L patients with ocular manifestations remain limited, with only five cases documented in the literature (Depienne et al., 2013; Di Bella et al., 2014; Giorgio et al., 2017; Guo et al., 2019; Xu et al., 2023). In this report, we present a novel homozygous pathogenic c.1382_1386del (p.P461Lfs*13) mutation identified in exon 13 of the *CLCN2* gene in a patient with CC2L. Additionally, a new and noteworthy sign, vacuole-like vitreoretinopathy, was observed in this patient. These findings expanded the genetic and clinical spectrum of CC2L, shedding further light on the ocular manifestations associated with this condition.

## Case presentation

**FIGURE 1:**
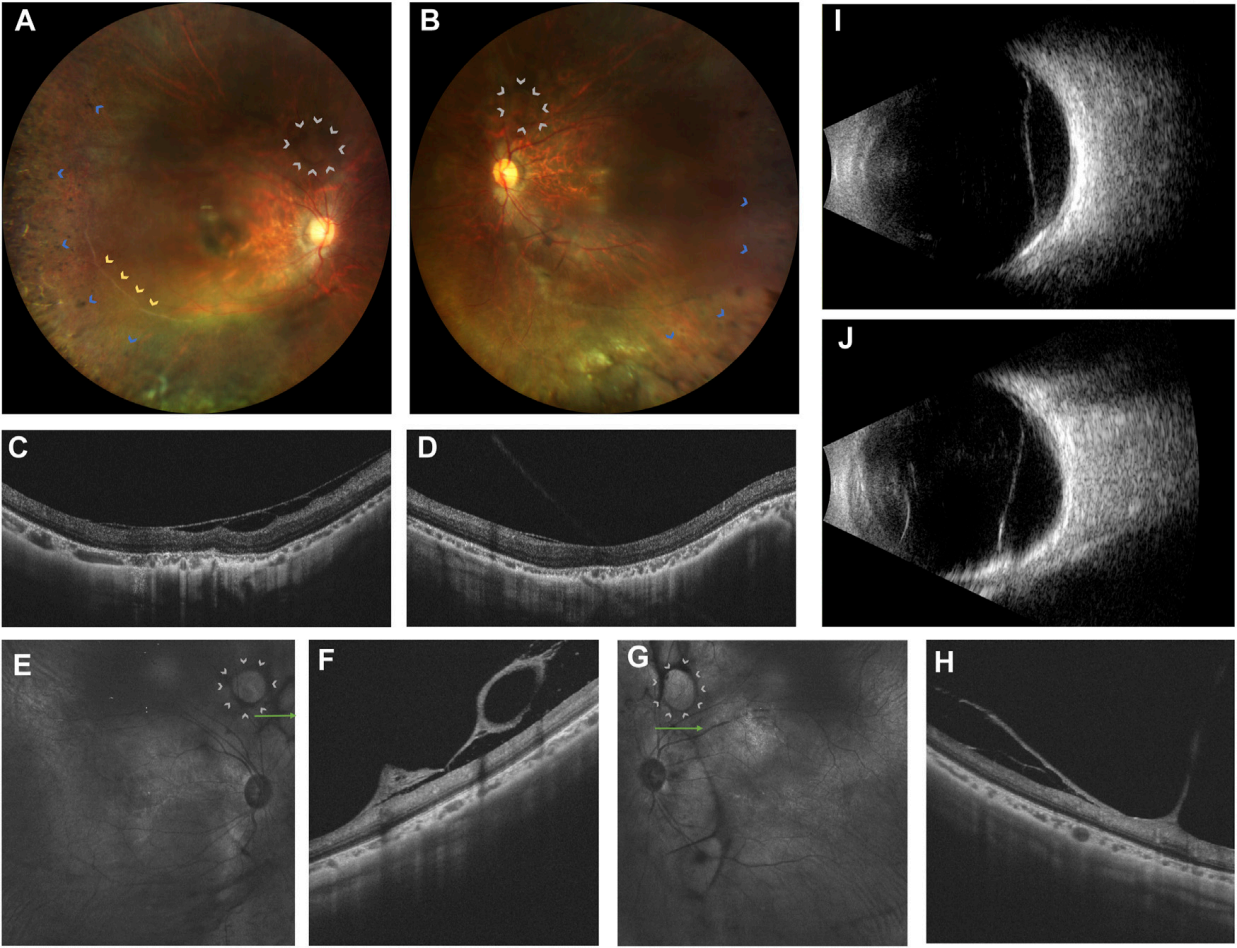
Ophthalmic multimodal imaging of the patient. **(A,B)** showed vacuole-like vitreoretinal fibro-cellular membrane (white arrows), vascular sheathing (yellow arrows), choroiretinal atrophy and bone spicule pigmentation (blue arrows) on fundus photograph. **(C,D)** showed epiretinal membrane, loss and disruption of the photoreceptor layer and choroidal atrophy. **(E–H)** showed hyperreflective vacuole-like vitreoretinal abnormalities B-scan optical coherence tomography and low reflectance of vacuole-like membranes on the near-infrared imaging. **(I,J)** showed a strong echo of a flat notch in the B-ultrasound.

A 20-year-old woman presented with bilateral blurred vision persisting for 2 years, worsening over the last 6 months. Initial suspicion of retinitis pigmentosa (RP) was noted 2 years ago. Her medical history was unremarkable, and there was no consanguinity between her parents. She had high myopia for 8 years, with -9D in the right eye and -7D in the left eye. Best corrected visual acuity was 0.82 logMAR in the right eye and 0.70 logMAR in the left eye. The intraocular pressure was unremarkable. Bilateral post-capsular cataract, pepper-like retinal pigmentation was noted in both eyes, with white retinal vascular sheathing in the sub-temporal regions of the right eye (Figures 1A, B). Swept-source optical coherence tomography (OCT) showed bilateral choroidal capillary atrophy, loss of the outer retinal layer, especially in the ellipsoid zone. (Figures 1C–H). Fundus fluorescence angiography (FFA) showed intense macular fluorescence and increased background fluorescence. Full-field electroretinography showed moderately reduced rod and cone responses. Humphrey perimetry revealed bilateral central and temporal field defects.

Furthermore, grayish-white vacuole-like or linear fibrous epiretinal thick membranes, adhered with the retina, predominantly in the periphery, were noted. OCT revealed it as a vacuole-like vitreoretinal adhesion. Magnified OCT showed the boundary and shape of this lesion clearly. Near-infrared imaging revealed low reflectance of the vacuole-like epiretinal membrane (Figures 1E–H). Thus, we name it as vacuole-like vitreoretinopathy.

**FIGURE 2:**
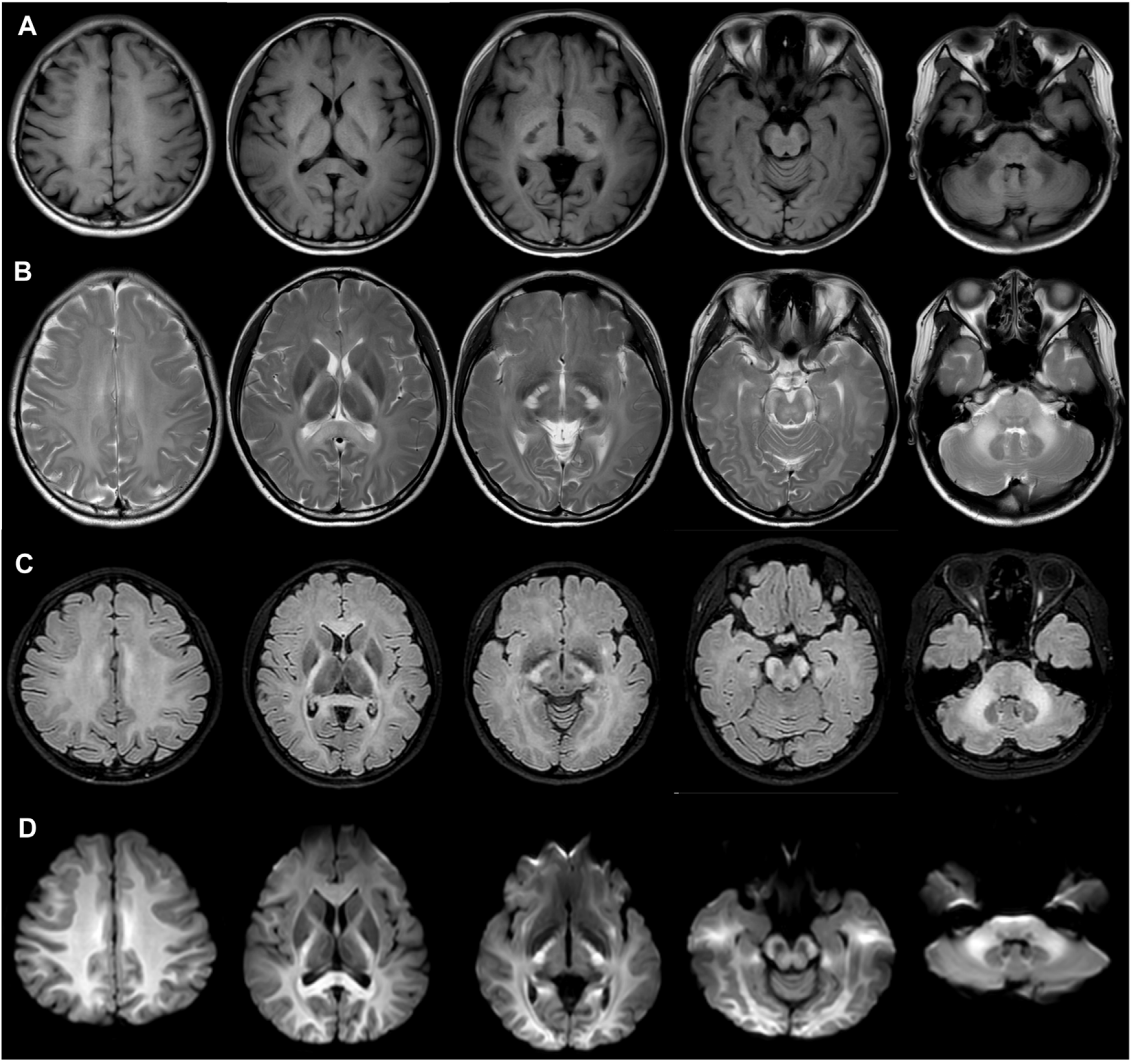
Magnetic resonance imaging showed confluent white matter abnormalities with hypointense T1-weighted **(A)** and hyperintense T2-weighted signals **(B)**, with the symmetrical involvement of the posterior limbs of the internal capsules, splenium of the corpus callosum, midbrain cerebral peduncles, pons, middle cerebellar peduncles. Fluid attenuated inversion recovery T2 **(C)** and diffusion-weight imaging revealed hyperintensity in the involved areas. **(D)**.

The patient did not exhibit any neurological symptoms such as headache, ataxia, poor motor ability, hearing impairment or loss, or memory decline. However, conventional magnetic resonance imaging (MRI) revealed confluent white matter abnormalities with hypointense T1-weighted and hyperintense T2-weighted signals, symmetrically involving the posterior limbs of the internal capsules, splenium of the corpus callosum, midbrain cerebral peduncles, pons, middle cerebellar peduncles (Figures 2A, B). Fluid-attenuated inversion recovery T2 and diffusion-weight imaging revealed hyperintensity in the affected areas (Figures 2C, D). The patient’s mother, father, and brother showed no abnormalities in their fundus (Supplementary Figure).

**FIGURE 3:**
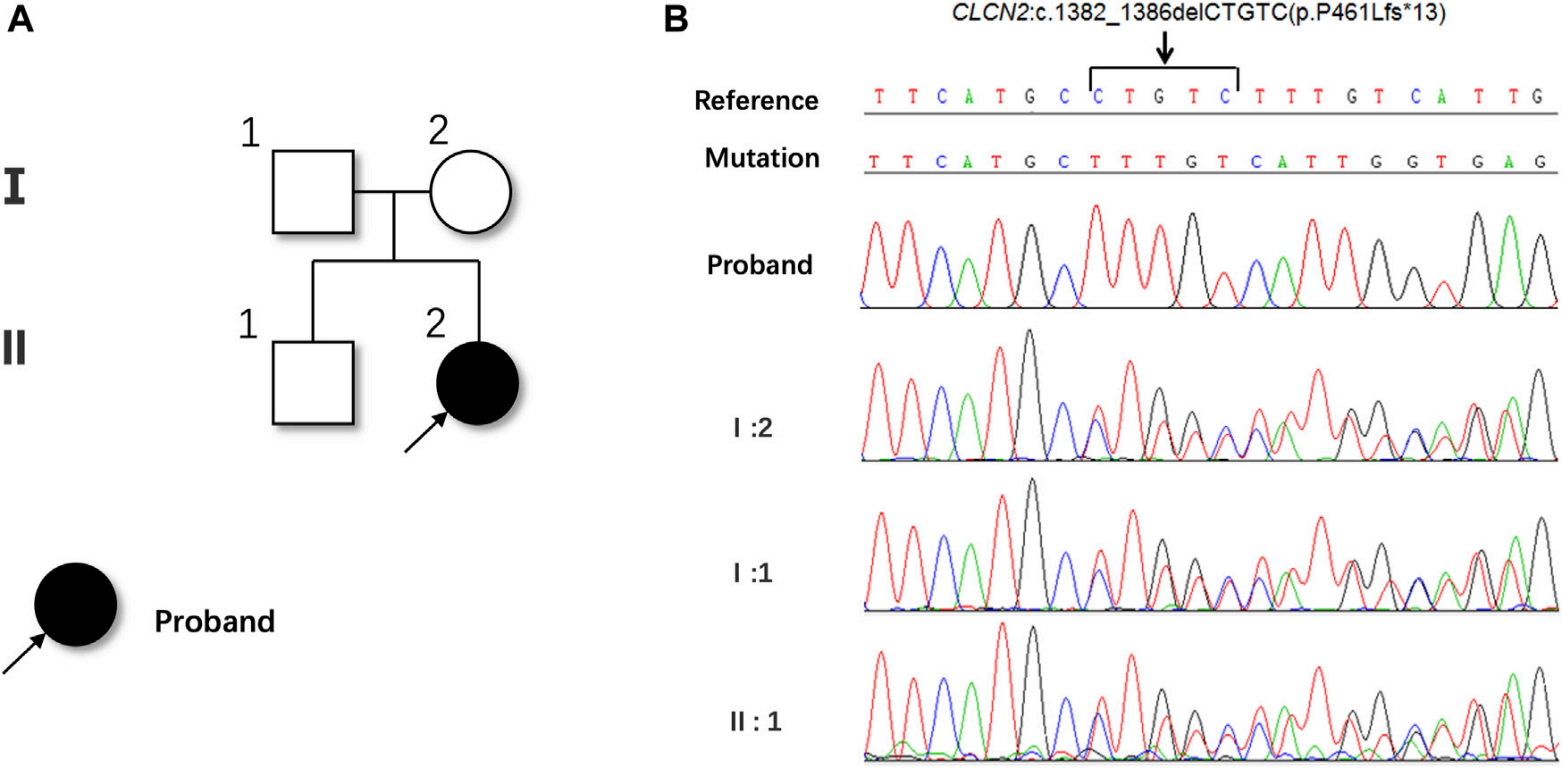
Pedigree **(A)** and sequencing chromatograms **(B)** of the family.

Whole exome sequencing (WES) was performed to unravel the genetic pathogenesis of this rare disease. Whole-exome sequencing revealed a novel homozygous c.1382_1386del (p.P461Lfs*13) mutation in the exon 13 of the *CLCN2* gene, resulting in a frameshift mutation that changed the 461th proline to leucine and caused premature termination of protein synthesis. This mutation was not found in the genetic database and was considered pathogenic. The patient’s mother, father and brother were identified as heterozygous carriers of this mutation (Figures 3A, B).

## Material and methods

### DNA extraction

Blood samples were collected from the proband and her unaffected family members. Genomic DNA was isolated as mentioned in our previous study (Tang et al., 2016).

### Whole exome sequencing

Whole exome sequencing (WES) was performed to unravel the genetic pathogenesis of this rare disease. Sequencing was performed on an Illumina NovaSeq (Illumina, Madison, WI, United States), with a read length of 2 × 150 bp. Single-nucleotide variants (SNVs) and insertions and deletions (InDels) were called by GATK4.0 HC with sequencing depth set to 100-fold. Strand NGS software version 2.0 (Strand Scientific Intelligence Inc., LA, United States) was used to set the sequencing reads to University of California Santa Cruz hg19 (2009.2). The minor allele frequency was defined as less than 0.03% in the public database Genome Aggregation Database (gnomAD, http://www.gnomad-sg.org, version 2.1). The Human Gene Mutation Database (HGMD; http://www.hgmd.cf.ac.uk/ac/index.php, 2020.4), dbSNP151 (http://www.ncbi.nlm.nih.gov/SNP/, build 151), ClinVar (https://www.clinicalgenome.org/data-sharing/clinvar/) and Exome Aggregation Consortium (ExAC, http://exac.broadinstitute.org/) were also used to identify the reported pathogenic variants. The pathogenicity of missense variants was further estimated by using SIFT (ensembl 66, released 2015.01 http://sift.jcvi.org), PolyPhen-2 (version 2.2.2, released v2.2.2, released 2012.02, http://genetics.bwh.harvard.edu/pph2/) (Adzhubei et al., 2010), Mutation Taster (data retrieved in 2015, http://www.mutationtaster.org) (Steinhaus et al., 2021), PROVEAN (version 1.1, ensembl 66, released 2015.01 http://provean.jcvi.org/index.php) (Choi and Chan, 2015) and CADD (version 1.6, http://cadd.gs.washington.edu/) (Rentzsch et al., 2021) online algorithms and via evolutionary conservation analysis. The classification of sequence variants was evaluated based on the ACMG standards and guidelines (Richards et al., 2015).

## Discussion

In this report, the case clinically and genetically confirmed CC2L, initially with blurred vision, without any neurological symptoms, with a homozygous pathogenic mutation in the *CLCN2* gene identified by WES. CC2L, a rare syndrome, manifests with a chronic course, predominantly occurring in females. Most patients with CC2L only have mild neurologic deficits. Specific changes in MRI are important clinical clues to support the diagnosis. Since *CLCN2*-knocked-out mice were first reported to exhibit brain and retinal abnormalities (Bösl et al., 2001; Blanz et al., 2007), many *CLCN2* variants have been identified in individuals with CC2L (Depienne et al., 2013; Di Bella et al., 2014; Hanagasi et al., 2015; Giorgio et al., 2017; Zeydan et al., 2017; Guo et al., 2019; Hoshi et al., 2019; Ngo et al., 2020; Ozaki et al., 2020; Parayil Sankaran et al., 2020). Among these individuals, only five of them exhibited ocular manifestations, including poor vision, optic neuropathy, visual field defects, retinoschisis, retinal atrophy, and normal fundi appearance with abnormal electroretinography and visual evoked potential. To date, we still know little about the ocular manifestation of CCL2.

The underlying mechanisms of *CLCN2*-related retinal degeneration remained elusive. Depienne et al. believe that the water-electrolyte imbalance of brain tissue caused by the loss of function in the *CLCN2* gene, causing osmotic intramyelination edema, is an important cause of this characteristic MRI change (Depienne et al., 2013). The *CLCN2*-knocked-out mice and *CNCL2* ^nmf240^ mice homozygotes both develop early-onset and severe photoreceptor degeneration, with only a single layer of photoreceptor cells remaining (Bösl et al., 2001; Edwards et al., 2010). Xu et al. found that the patient induced pluripotent stem cells (iPSC)-derived retinal pigment epithelium (RPE) cells carrying *CLCN2* mutations exhibited dysfunctions of ClC-2 chloride channels and outer segment phagocytosis. Repaired by the CRISPR-CaS9 system, the aforementioned dysfunctions were rescued. The findings reported by Xu et al. suggested that the retinal degeneration caused by *CLCN2* mutation may be due to RPE dysfunction. Given that, the overaccumulation of metabolic wastes may cause degeneration of retinal photoreceptors (Xu et al., 2023). However, the pathogenic mechanisms should be further investigated, due to the varied phenotypes in different variants of the *CLCN2* gene.

We verified that the *CLCN2* gene is associated with retinal degeneration, which should be routinely added to the clinical retinopathy genetic sequencing panel. Guo and Xu et al. reported a Chinese 38-year-old female exhibiting a similar ocular appearance to our case, including bilateral cataract, myopia, peripheral retinal pigmentation, retinal atrophy, and loss and disruption of the outer retina on OCT (Xu et al., 2023). Both our case and Guo’s case indicated that *CLCN2*-related retinal degeneration is an autosomal-recessive inherited retinal dystrophy with systemic abnormalities. Furthermore, we offered detailed descriptions of the novel ocular manifestation of vacuole-like vitreoretinopathy, which is displayed by fundus photography, and infrared- and B-scan OCT. The association between *CLCN2* mutation and the occurrence of multiple vacuole-like vitreoretinal adhesion is not clear. We speculate the depletion of the ClC-2 may impair the transport and alter the ionic environment of glial cells and hyalocytes, causing the formation of fibro-cellular proliferation. In addition, inflammation may be another contributor to the formation of vacuole-like vitreoretinopathy (Fujiwara et al., 2016). Since our patient shares some features with RP, the mechanism of the epiretinal membrane formation in RP may hint us the underlying pathway of vacuole-like vitreoretinopathy in CCL2-related retinopathy. Fujiwara et al. retrospectively reviewed a relatively large cohort of RP patients and found that inflammation is a significant cause in association with the presence of epiretinal membrane. Nevertheless, our case showed thicker membranes, more multiple sites of vitreoretinal adhesions and a specifically vacuole-like shape of epiretinal proliferation compared to epiretinal membranes in RP (Fujiwara et al., 2016; Tan et al., 2021). Our case also showed some similarities with Wagner vitreoretinopathy. Wagner vitreoretinopathy, associated with *VCAN* gene, is characterized by an optically empty vitreous with avascular pre-retinal vitreous strands and veils (Meredith et al., 2007; Li et al., 2020). Progressive chorioretinal atrophy, presenile cataract, mild to moderate myopia, ectopic fovea, and retinal detachment were commonly recorded (Meredith et al., 2007). However, the preretinal vitreous strands and veils were often observed in the mid-peripheral retina (Li et al., 2020), which is different from our patient. In total, both the specific genetic background and inflammation probably contribute to the formation of vacuole-like vitreoretinopathy, though other factors like age and sex cannot be excluded. More experiments and case series studies of CCL2-related retinopathy should be conducted to explore and validate the relationship between the *CLCN2* gene and retinal degeneration.

We reported the first CC2L case that initially presented with only ocular symptoms, not exhibiting any neurological symptoms. The ophthalmic findings could provide important evidence for early detection in patients with CC2L but without obvious neurological symptoms. When facing patients initially presented with ocular manifestations, MRI and WES are helpful for early diagnosis of CC2L. However, due to the limited case number and retrospective nature, the diagnostic value of vacuole-like vitreoretinopathy for CC2L needs to be validated in more CC2L patients.

In conclusion, we expanded the genotypic and phenotypic spectrum of the *CLCN2* gene. We identified a novel homozygous c.1382_1386del (p.P461Lfs*13) mutation in the *CLCN2* gene and found a novel ocular feature of vacuole-like vitreoretinopathy. Fundus complications should be considered during screening, diagnosis, and treatment. Our report verified that *CLCN2*-related retinopathy may be a kind of inherited retinal dystrophy. The identified ophthalmic features may serve as crucial indicators for early diagnosis in individuals with CC2L, especially in the absence of evident neurological symptoms.

## Data Availability

The datasets for this article are not publicly available due to concerns regarding participant/patient anonymity. Requests to access the datasets should be directed to the corresponding author.

## Abbreviations

CC2L, CLCN2-related leukoencephalopathy; FFA, fundus fluorescence angiography, MRI, magnetic resonance imaging; SS-OCT, swept-source optical coherence tomography; WES, whole-exome sequencing.
