# Supplementary figures and images for: Case report: A frameshift mutation in CLCN2-related leukoencephalopathy and retinopathy
Source: Front Genet. 2023 Nov 9;14:1278961. doi: 10.3389/fgene.2023.1278961 (PMC10665509; doi:10.3389/fgene.2023.1278961)

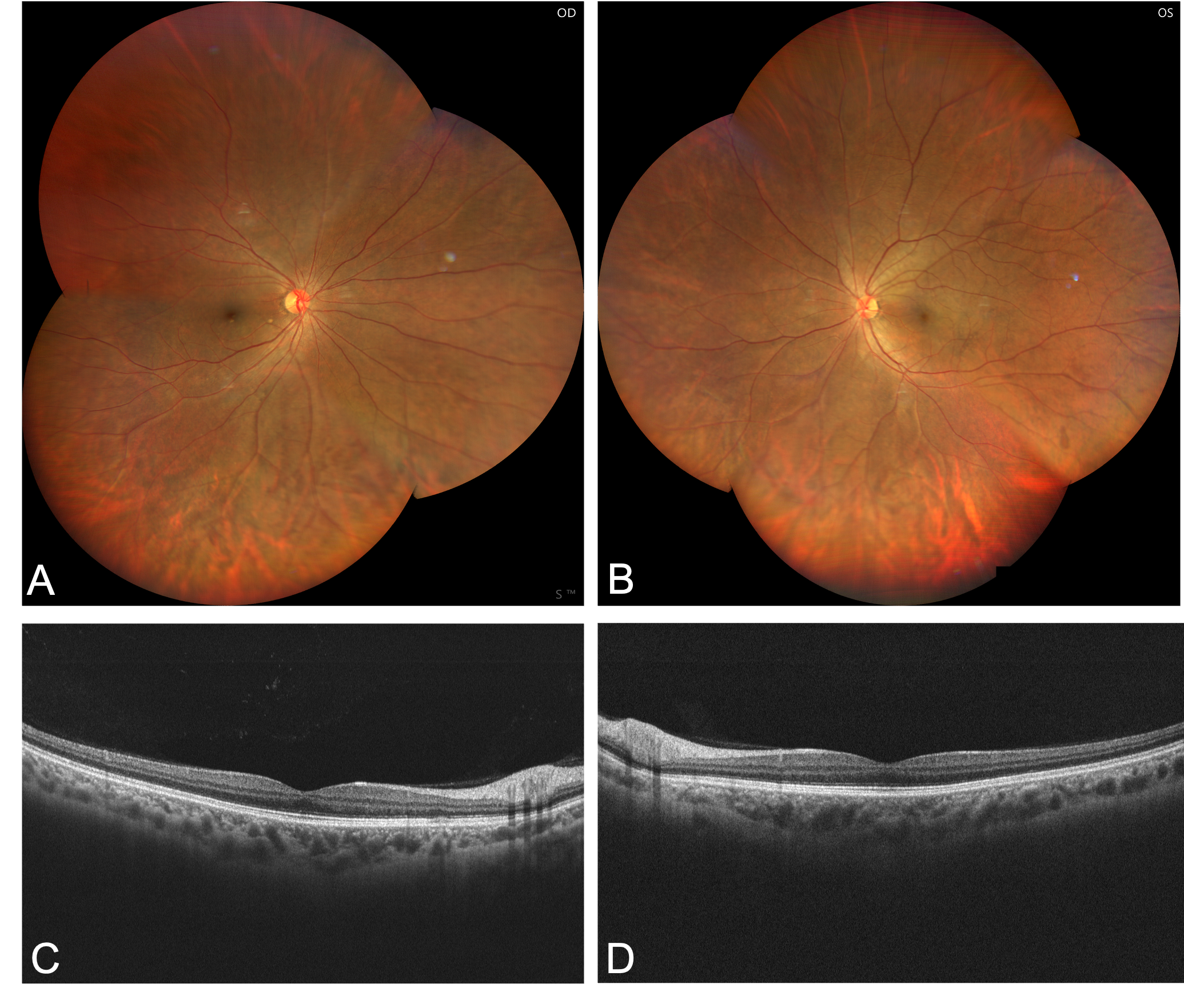

Supplement: Supplementary file 1 [file Image1.JPEG]
